# Schistocytes Evaluation in Iron Deficiency: An Assessment Adopted From ICSH Nomenclature Guideline

**DOI:** 10.1155/anem/9990762

**Published:** 2025-07-21

**Authors:** Tri Ratnaningsih, Galih R. Martani, Dhia C. Putri, Usi Sukorini

**Affiliations:** ^1^Department of Clinical Pathology and Laboratory Medicine, Faculty of Medicine, Public Health and Nursing, Universitas Gadjah Mada, Yogyakarta, Indonesia; ^2^Clinical Pathology Specialist Program, Faculty of Medicine, Public Health and Nursing, Universitas Gadjah Mada, Yogyakarta, Indonesia; ^3^PKU Muhammadiyah Yogyakarta Hospital, Yogyakarta, Indonesia

**Keywords:** ferritin, iron deficiency, microcytic, schistocyte

## Abstract

**Introduction:** The diagnostic validity of schistocyte count in diagnosing iron deficiency (ID) in the microcytic population is critical. The purpose of this study is to identify the correlation between schistocyte count and iron parameters and the performance of schistocyte count in diagnosing ID in the microcytic population.

**Methods:** Out of 805 general checkup participants, 65 subjects consisting of 17 males and 48 females aged 18–56 years had Mean Corpuscular Value (MCV) < 80 fL. Serum ferritin examination showed 25 patients with ID and the other 40 subjects without ID.

**Result:** There was a significant difference in the schistocyte count between the two groups (*p* < 0.001). Correlation analysis obtained a significant relationship between schistocyte count and serum ferritin (*r* = −0.67, *p* < 0.001). The receiver operating characteristic (ROC) curve analysis provided an area under the curve (AUC) of schistocyte count of 0.827, with a sensitivity of 80% and specificity of 75% for a cutoff of ≥ 0.75%.

**Conclusion:** Schistocyte count has a significant correlation with iron parameters and can be used as a marker for ID in the microcytic population. Health facilities that do not have access to iron parameters examination can perform a schistocyte count.

## 1. Introduction

Anemia is a worldwide public health issue affecting approximately a quarter of the world's population. Among many different anemia causes, iron deficiency anemia (IDA) is the most widespread [1]. Anemia can be caused by nutritional deficiencies (iron, folate, vitamin B12, and vitamin A), acute and chronic inflammation, parasitic infections, and inherited or acquired disorders that can affect the synthesis of hemoglobin, red blood cell (RBC) production, or RBC survival [2]. In the latest study about global burden of IDA, developed countries showed greater improvement with 25.7% reduction compared with only 11.5% reduction in developing country [3]. In Indonesia, iron deficiency (ID) is responsible for most cases of anemia. Many of the adverse effects caused by anemia include having a baby with low birth weight [4], maternal infectious morbidities [5], developmental delay in children [6], cognitive disorders and intellectual at school age [7, 8], and decreased productivity in adulthood [9, 10].

In ID, the microcytic erythrocytes found are caused by a decrease in the concentration of hemoglobin. While iron composes most of the hemoglobin structure, the erythrocytes formed under ID conditions will present smaller (microcytic) and pale (hypochromic) in morphology [11]. The most common parameters used for iron status measurements are transferrin saturation and serum ferritin. Unfortunately, the iron biochemical parameters as the gold standard to detect ID are still relatively costly and not affordable enough to be used in daily practice at peripheral health facilities [12, 13]. In Indonesia, government-owned health facilities at the subdistrict level and even type D hospitals do not necessarily have the photometer or reagent equipment needed to check serum iron and ferritin. Hb examination using the Sahli method is still common, so microscopy will be very helpful because microscopes are available in all health laboratory services. Due to this condition, a simple alternative assessment tool for laboratory facilities located in rural and remote areas is considered necessary. Some parameters of erythrocytes have been widely investigated concerning their relation to iron status [14]. In addition to elliptocytes, ovalocytes, target cells, and hypochromic, which have been known to be the most common in IDA, it turns out that fragment cells (schistocytes) have also been reported in ID [15].

Schistocyte (from Greek schistos for “divided” and kytos for “hollow” or “cell”) is a fragmented part of a RBC that can be seen at peripheral blood smear as an irregularly shaped body with two pointed ends. Schistocyte occurs due to obstacles in blood vessels, such as fibrin clots, artificial heart valves, or other blood vessels' obstacles. Schistocyte count is considered normal at the level of < 0.5% [16]. RBC fragments represented as schistocytes can occur on microscopic examination of peripheral blood smears due to several conditions [13].

In ID, erythrocytes with poikilocytosis morphology, such as schistocytes, have a shorter life span and membrane stiffness. The iron that builds up hemoglobin is also the primary feature of the erythrocyte structure. If the iron levels are decreased, there will be an increase in the surface–volume ratio of erythrocytes. This change causes erythrocytes easy to break when passing through capillaries or turbulence, causing erythrocyte fragments to be formed in the bloodstream [17].

According to researchers' knowledge, studies on RBC fragment as an alternative parameter to detect ID in Indonesia are still limited. This study aims to determine the correlation between schistocyte count and iron parameters and schistocyte count performance in diagnosing ID in the microcytic population.

## 2. Materials and Methods

This research is a cross-sectional observational study with a population consisting of healthy participants for thalassemia trait screening. The participants who agreed becoming research subjects were drawn 4 mL of venous blood samples in a tube containing EDTA and 4 mL blood with a plain tube for iron analysis. Inclusion criteria included all male and female screening participants who had microcytic erythrocyte morphology (mean cospucular valuer (MCV) < 80 fL). Subjects were excluded if they were pregnant.

Complete blood count (CBC) data were obtained from an automated hematology analyzer (Advia 2120, Siemens Medical Solutions Diagnostics, Tarrytown, NY). We included 68 individual participants who had microcytic hypochromic (MCV < 80 fL and MCH < 28 pg) erythrocyte morphology who were then proceeded to iron analysis using Cobas 6000® analyzer series (Roche Diagnostics Corporation, Indianapolis, USA). After we performed a serum ferritin examination, we selected 43 ID and 25 non-ID cases. Subjects were considered iron deficient if serum ferritin levels were < 12 ng/mL. Blood smears were prepared with a Wright–Giemsa stain. Schistocytes per 1000 RBCs were calculated in oil immersion fields, using Microscope Olympus CX23 (Olympus Corporation, Tokyo, Japan). Based on the International Council for Standardization in Haematology (ICSH) 2015 guideline morphology criteria, schistocytes are characterized as cells smaller than intact red cells and can have the shape of fragments with sharp angles and straight borders, small crescents, helmet cells, or keratocytes. Morphologic features were assessed in a blinded manner, without knowledge of the iron studies. Schistocyte count was done with good reliability results between two examiners (*r* = 0.944 and *p* = 0.016).

The estimated sample size is calculated based on the single proportion sample size formula in diagnostic research with sensitivity value output [18].

(1) $$\begin{array}{c} n=\frac{{\left(Z\alpha \right)}^{2}p\left(1-p\right)}{d^{2}P}, \end{array}$$

where *n* = minimum sample size for diagnostic test, *p* = desired sensitivity at cutoff value, *d* = width deviation of the *p* value that is still acceptable, which is ± 0.1, *Zα* = error rate is set at 5% (95% CI), *Zα* = 1.96, and *P* = prevalence of IDA (55%)

The result of the minimum sample size is 62.86.

The normality test for hematology parameter, schictocytes, and iron status was the Kolmogorov–Smirnov. This resulted to see data that are distributed normal or not, then followed by the mean difference test using the Mann–Whitney *U* test for non-normal data distribution and Student's *t*-test for normal data distribution, as shown in Table 1. Scatter plots using the Spearman correlation test were used to evaluate the relationship between two variables. Receiver operating characteristic (ROC) curves were drawn to determine the optimal cutoff of the schistocyte ability as an ID marker. The area under the curve (AUC), the sensitivity, specificity, positive predictive value (PPV), and negative predictive value (NPV) of selected cutoff points were calculated.

All statistical analyses were performed using SPSS Statistics for Windows, Version 25 (SPSS Inc, Chicago, Illinois). A two-sided *p* value of 0.05 or less was considered statistically significant. This study has received ethical approval from the FK-KMK UGM Ethics Committee (no: KE/FK/0552/EC/2019). All examinations were carried out at the Clinical Pathology Laboratory, FK-KMK UGM, and Clinical Laboratory Unit, RSUP Dr. Sardjito.

## 3. Result

**Figure 1:**
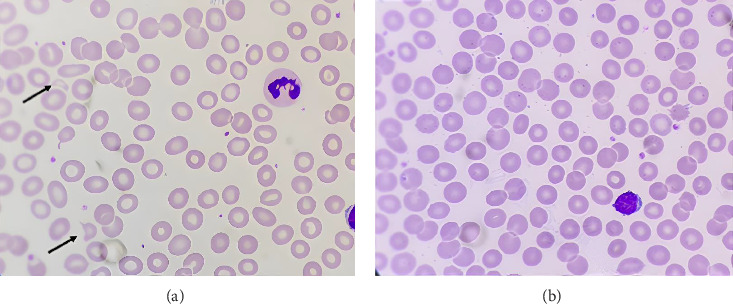
Blood picture of schistocyte in iron deficiency subject. (a) Iron deficiency subject. Schistocyte as a fragment and triangular found in the smear. (b) Microcytic noniron deficiency subject. No fragmented cells were found in the smear.

Of the 805 general checkup participants, 65 subjects had MCV < 80 fL. These microcytic subjects consisted of 17 males and 48 females with 18–56 years. The subjects were then grouped into ID and non-ID based on serum ferritin levels. In this study, 25 ID subjects and 40 non-ID subjects were obtained.  Figure 1 shows the peripheral blood smear results in both microcytic subjects with ID, which has fragmented RBCs, and the ones without ID, which has no fragmented cells. The erythrocyte index results from the CBC and iron status of all microcytic subjects are shown in Table 1.

**Figure 2:**
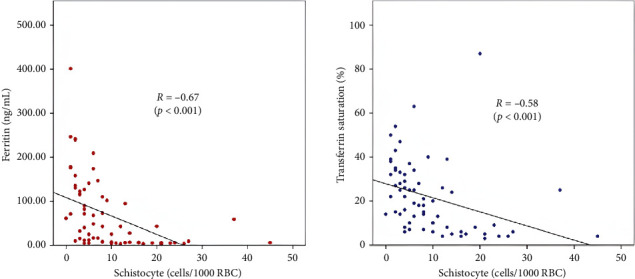
Schistocyte count and iron parameters correlation in the microcytic population.

There was a significant difference in the schistocyte count between the two groups (*p* < 0.05). Spearman correlation test in Figure 2 shows a correlation between schistocyte count and serum ferritin level (*r* = −0.67 and *p* < 0.001), as well as between schistocyte count and transferrin saturation (*r* = −0.58 and *p* < 0.001).

**Figure 3:**
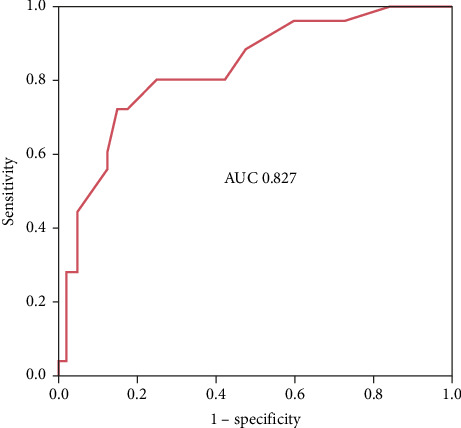
ROC curve and diagnostic performance of schistocyte.

In the ROC curve analysis of schistocyte count with serum ferritin as the gold standard, an AUC of 0.827 was obtained (Figure 3). The cutoff for schistocyte count was 0.75% with a sensitivity of 80%, specificity of 75%, PPV of 61%, NPV of 86%, positive likelihood ratio of 2.67, and negative likelihood ratio of 0.286.

## 4. Discussion

Based on the subjects' characteristics, females were likely to have microcytic RBCs than males, with an age range of 18–56 years old. In other studies, it is found that hypochromic microcytic anemia is more common in premenopausal females because they lose blood with each menstrual cycle. After the female population, preschool-aged children suffer the most from anemia because of a lack of iron in their primary diet. The male population is usually resistant to anemia due to circulating testosterone levels. Among 65 microcytic subjects, 26% were males, a slightly higher number than the percentage of adult males who are globally afflicted with anemia, which is 12.7% [19, 20]. This study's limitation is that it does not include the age of children as research subjects; the study used general checkup participants. Therefore, the WHO does not distinguish between ferritin cutoff value in determining an ID in children and adults; then, these results may apply to the pediatric population. However, it is better if similar research uses children as the subject to prove the above assumptions.

There was a significant difference in the schistocyte count between ID subjects than non-ID subjects (*p* < 0.05). Microcytic subjects with ID had fragmented RBCs, while the ones without it had none. Schistocyte, a fragmented red cell, usually occurs in cytoskeletal RBC abnormalities, such as acquired and inherited RBC disorders in association with marked anisopoikilocytosis [21]. A study on direct red cell membrane deformability measurements indicated that ID might increase membrane rigidity, causing reduced RBC deformability, which produced fragmented cells [20]. Another study also stated that RBC fragments are most numerous in diseases with marked anisopoikilocytosis, such as IDA [22].

Reduced hemoglobin levels with low MCV may suggest IDA, indicating red cell morphology and other ancillary investigation for ID. Further tests such as serum ferritin and total iron-binding capacity (TIBC) help to differentiate microcytic anemia. For instance, low serum ferritin is expected in ID [23, 24]. This statement was confirmed in our study, where there was a statistically significant inverse relationship between schistocyte count and serum ferritin levels in the correlation analysis of this study (*r* = −0.67; *p* < 0.001), indicating an association of higher schistocyte count with lower serum ferritin levels leading to the direction of ID.

The AUC obtained from the ROC curve analysis (0.827) has sufficient diagnostic ability, where the minimum AUC is equal to or more than 0.7. As we know, in general, an AUC of 0.5 suggests no discrimination (i.e., ability to diagnose patients with and without the disease or condition based on the test), 0.7–0.8 is considered acceptable, 0.8–0.9 is considered excellent, and more than 0.9 is considered outstanding [25]. This result indicates that schistocyte count has a sufficient ability to mark ID in the microcytic population.

One has to distinguish the underlying disease that shows schistocytes on the peripheral blood picture because there are other conditions in which schistocytes are found in the peripheral bloodstream. One of them is syndromes of thrombotic microangiopathic anemia (TMA), such as thrombotic thrombocytopenic purpura and hemolytic uremic syndrome. In TMA, schistocytes are not accompanied by microcytic, accompanying low platelets, and the absence of additional severe RBC morphological abnormalities. In ID, anemia is not accompanied by thrombocytopenia. Instead, thrombocytosis often occurs, and the morphological abnormalities of RBC have many variations [16]. In TMA cases, schistocyte count and grading are recommended and may be valuable when schistocytes are the dominant feature (add with polychromasia, nucleated red blood cell (NRBC), and thrombocytopenia) for the diagnosis and follow-up of microangipathy hemolitic anemia (MAHA). Schistocyte is termed few or 1+ if the number is < 1%; moderate or 2+ if 1%–2%; and many or 3+ if > 2% [26]. Meanwhile, in another reference, schistocyte is termed occasional if it is less 1%; 1+ if 1%–3%; 2+ if 3%–6%; and 4+ if it is more than 12% [21].

ICSH recommends grading schistocytes. A schistocyte count and grading are recommended and may be valuable when schistocytes are the dominant feature (add with polychromasia, NRBC, and thrombocytopenia) for the diagnosis and follow-up of MAHA. Schistocytes will have a greater significance at lower percentage numbers than other abnormalities. The designation for 1+ (few/rare) is reserved only for schistocytes, as the observation, even in small numbers, is clinically significant [23]. Response to IDA treatment can be made by assessing the reduced number of schistocyte found in peripheral blood. The clinical significance of schistocyte is smaller than that of other cell types so that careful examination of 1000 erythrocyte cells is necessary when following up monitoring of iron therapy response.

Any positive smear findings represent relevant clinical information that must be described in CBCs as per local consensus indicating the morphological changes and cell percentages relevant for the diagnosis and monitoring of patients [23].

Due to a small sample size, there will be some difficulty to generalize the result of this study to wider population. Yet, this result can be an illustration about how to do schistocytes evaluation in ID as a preliminary study. Further studies in larger number are needed for its use in generalizability.

## 5. Conclusion

Schistocyte count has a significant correlation with iron parameters and can be used as a marker for ID in the microcytic population. However, studies with subjects who are not only the microcytic population are required to prove schistocyte's diagnostic power to identify an ID in the general population. This study only used 65 subjects as a preliminary study. Future work can use more subjects to obtain more convincing results.

## Figures and Tables

**Table 1:** The characteristic of a hematology profile and schistocyte count.

Parameter	Iron deficiency (*n* = 25)	Noniron deficiency (*n* = 40)	*p* value
Hemoglobin (g/dL)	10.48 ± 1.43	13.19 ± 1.65	< 0.001^a∗^
Hematocrit (%)	34.98 ± 3.20	41.94 ± 4.61	< 0.001^a∗^
Erythrocyte count (× 10^6^ cells/μL)	4.96 ± 0.41	5.78 ± 0.62	< 0.001^a∗^
Mean corpuscular volume (MCV) (fL)	73.5 (50–79)	74.7 (58–80)	0.237^b∗^
Mean corpuscular hemoglobin (MCH) (pg)	21.90 (13–26)	23.8 (17–25)	0.018^b∗^
Mean corpuscular hemoglobin concentration (MCHC) (%)	29.83 ± 1.84	31.4 ± 1.20	< 0.001^a∗^
Schistocyte (cells/1000 RBC)	13.00 (2–45)	4.00 (0–37)	< 0.001^b∗^

^a^Student's *t*-test.

^b^Mann–Whitney U test.

^∗^Statistically significant (*p* < 0.05).

## Data Availability

The datasets used and/or analyzed during this study are available from the corresponding author upon reasonable request.

## References

[1] Alzaheb R. A., Al-Amer O. (2017). The Prevalence of Iron Deficiency Anemia and Its Associated Risk Factors Among a Sample of Female University Students in Tabuk, Saudi Arabia. *Clinical Medicine Insights: Women’s Health*.

[2] Lee S., Son Y., Hwang J. (2025). Global, Regional and National Burden of Dietary Iron Deficiency From 1990 to 2021: A Global Burden of Disease Study. *Nature Medicine*.

[3] Who (2011). *Vitamin and Mineral Nutrition Information System*.

[4] Mansbridge J. (1998). Skin Substitutes to Enhance Wound Healing. *Expert Opinion on Investigational Drugs*.

[5] Urrechaga E. (2008). Discriminant Value of % Microcytic/% Hypochromic Ratio in the Differential Diagnosis of Microcytic Anemia. *Clinical Chemistry and Laboratory Medicine*.

[6] Lanzkowsky P. (2016). *Iron-Deficiency Anemia*.

[7] Grote Beverborg N., Klip I. T., Meijers W. C. (2018). Definition of Iron Deficiency Based on the Gold Standard of Bone Marrow Iron Staining in Heart Failure Patients. *Circulation: Heart Failure*.

[8] Brugnara C. (2002). A Hematologic “Gold Standard” for Iron-Deficient States?. *Clinical Chemistry*.

[9] Barcellini W., Fattizzo B. (2015). Clinical Applications of Hemolytic Markers in the Differential Diagnosis and Management of Hemolytic Anemia. *Disease Markers*.

[10] Constantino B. T. (2015). Reporting and Grading of Abnormal Red Blood Cell Morphology. *The International Journal of Literary Humanities*.

[11] Yip R., Mohandas N., Clark M. R., Jain S., Shohet S., Dallman P. (1983). Red Cell Membrane Stiffness in Iron Deficiency. *Blood*.

[12] Czyzewska E., Kujawiak A., Bobilewicz D., Szarpak L., Czyzewski L. (2018). The Assessment of the Utility of Novel Peripheral Blood Morphology Parameters, Including Reticulocytic, in the Diagnosis of Iron Deficiency and Sideropenic Anemia. *Postępy Nauk Medycznych*.

[13] Whitney M. S. (2012). Schistocytes. *Clinical Veterinary Advisor: The Horse*.

[14] Schapkaitz E., Raburabu S. (2018). Performance Evaluation of the New Measurement Channels on the Automated Sysmex XN-9000 Hematology Analyzer. *Clinical Biochemistry*.

[15] Chaundry H. S., Kasarla M. R. (2020). Microcytic Hypochromic Anemia. *StatPearls*.

[16] Alvarez-Uria G., Naik P. K., Midde M., Yalla P. S., Pakam R. (2014). Prevalence and Severity of Anaemia Stratified by Age and Gender in Rural India. *Anemia*.

[17] Ben-Ezra J. (2007). Clinical Characteristics of Patients with a RBC Fragment Flag on the Advia® 2120 Automated Hematology Analyzer. *Blood*.

[18] Lemeshow S., Jr D. W. H., Klar J., Lwanga S. K. (1990). *Erythrocyte*.

[19] Adeyowin A. S., Adeyemi O., Davies N. O., Ogbenna A. A. (2019). *Erythrocyte Morphology and Its Disorders*.

[20] Harrington A. M., Ward P. C. J., Kroft S. H. (2008). Iron Deficiency Anemia, β-Thalassemia Minor, and Anemia of Chronic Disease: A Morphologic Reappraisal. *American Journal of Clinical Pathology*.

[21] Kushwaha R., Singh U., Gupta C., Kumar A. (2013). An Analytical Study on Peripheral Blood Smears in Anemia and Correlation With Cell Counter Generated Red Cell Parameters. *J Appl Hematol.*.

[22] Palmer L., Briggs C., Mcfadden S. (2015). ICSH Recommendations for the Standardization of Nomenclature and Grading of Peripheral Blood Cell Morphological Features. *The International Journal of Literary Humanities*.

[23] Comar S. R., Malvezzi M., Pasquini R. (2017). Evaluation of Criteria of Manual Blood Smear Review Following Automated Complete Blood Counts in a Large University Hospital. *Revista Brasileira de Hematologia e Hemoterapia*.

[24] Ratnaningsih T., Martani G. R., Putri D. C., Sukorini U. (2020). Schistocytes Evaluation in Iron Deficiency: An Assessment Adopted From ICSH Nomenclature Guideline. *International Journal of Hematology*.

[25] Mandrekar J. N. (2010). Receiver Operating Characteristic Curve in Diagnostic Test Assessment. *Journal of Thoracic Oncology*.

